# Ventilator-associated pneumonia in neonates: the role of point of care lung ultrasound

**DOI:** 10.1007/s00431-020-03710-8

**Published:** 2020-06-26

**Authors:** Nora Tusor, Angela De Cunto, Yousef Basma, John L. Klein, Virginie Meau-Petit

**Affiliations:** 1 Neonatal Intensive Care Unit, Chelsea and Westminster Hospital NHS Foundation Trust, 369 Fulham Road, London, SW10 9NH UK; 2 Neonatal Intensive Care Unit, Evelina London Children’s Hospital, Guy’s and St Thomas’ NHS Foundation Trust, St Thomas’ Hospital, North Wing 6th floor, Westminster Bridge Road, London, SE1 0EH UK; 3 Neonatal Transfer Service London, Royal London Hospital, Whitechapel Road, London, E1 1FR UK; 4 Department of Infectious Diseases, Guy’s and St Thomas’ NHS Foundation Trust, St Thomas’ Hospital, North Wing 2nd floor, Westminster Bridge Road, London, SE1 0EH UK

**Keywords:** Neonatal, Preterm, Ventilator associated pneumonia, Lung ultrasound

## Abstract

No consensus exists regarding the definition of ventilator-associated pneumonia (VAP) in neonates and reliability of chest X-ray (CXR) is low. Lung ultrasound (LU) is a potential alternative diagnostic tool. The aim was to define characteristics of VAP in our patient population and propose a multiparameter score, incorporating LU, for VAP diagnosis. Between March 25, 2018, and May 25, 2019, infants with VAP were identified. Clinical, laboratory and microbiology data were collected. CXRs and LU scans were reviewed. A multiparameter VAP score, including LU, was calculated on Day 1 and Day 3 for infants with VAP and for a control group and compared with CXR. VAP incidence was 10.47 episodes/1000 ventilator days. LU and CXR were available for 31 episodes in 21 infants with VAP, and for six episodes in five patients without VAP. On Day 1, a VAP score of > 4, and on Day 3 a score of > 5 showed sensitivity of 0.94, and area under the curve of 0.91 and 0.97, respectively. AUC for clinical information only was 0.88 and for clinical and CXR 0.85.

*Conclusion*: The multiparameter VAP score including LU could be useful in diagnosing VAP in neonates with underlying lung pathology.

**Table:** 

**What is Known:** • *Ventilator associated pneumonia (VAP) is common in infants on the neonatal unit and is associated with increased use of antibiotics, prolonged ventilation and higher incidence of chronic lung disease.* • *Commonly used definitions of VAP are difficult to apply in neonates and interpretation of chest X-ray is challenging with poor inter-rater agreement in patients with underlying chronic lung disease.*	
**What is New:** • *The multiparameter VAP score combining clinical, microbiology and lung ultrasound (LU) data is predictive for VAP diagnosis in preterm infants with chronic lung disease.* • *LU findings of VAP in neonates showed high inter-rater agreement and included consolidated lung areas, dynamic bronchograms and pleural effusion.*

**What is Known:**

• *Ventilator associated pneumonia (VAP) is common in infants on the neonatal unit and is associated with increased use of antibiotics, prolonged ventilation and higher incidence of chronic lung disease.*

• *Commonly used definitions of VAP are difficult to apply in neonates and interpretation of chest X-ray is challenging with poor inter-rater agreement in patients with underlying chronic lung disease.*

**What is New:**

• *The multiparameter VAP score combining clinical, microbiology and lung ultrasound (LU) data is predictive for VAP diagnosis in preterm infants with chronic lung disease.*

• *LU findings of VAP in neonates showed high inter-rater agreement and included consolidated lung areas, dynamic bronchograms and pleural effusion.*

## Introduction

Ventilator-associated pneumonia (VAP) is the second most common cause of antibiotic use on the neonatal intensive care unit (NICU) [1]. VAP is associated with higher incidence of bronchopulmonary dysplasia, prolonged mechanical ventilation and hospital stay [2].

Definition of VAP is challenging in neonates with no international consensus [3]. In adults, VAP is generally defined as a nosocomial lower airway infection in intubated patients with onset after more than 48 h of invasive mechanical ventilation [4]. In neonates, frequently applied definitions from Centres for Disease Control and Prevention (CDC) and European Centre for Disease Prevention and Control (ECDC) are difficult to apply mainly due to the absence of specific clinical and laboratory findings. Several modified criteria have been developed. However, comparison of agreement between four diagnostic criteria showed only moderate agreement [5].

CDC and ECDC criteria require chest X-ray changes as one of the main criteria for VAP diagnosis. However, interpreting radiographic changes in patients with underlying lung pathology and on mechanical ventilation can be challenging and comorbidities such as chronic lung disease (CLD) can obscure radiographic evidence of VAP [6, 7]. Reliability of chest X-ray interpretation has been shown to be low for neonatal VAP [5].

Lung ultrasound (LU) is increasingly used in neonates to diagnose lung pathologies [8–11]. LU has been shown to be equivalent to chest X-ray for diagnosis of respiratory distress syndrome in infants [8, 9] and is highly accurate for the diagnosis of pneumonia in children [10, 11]. Multiparameter systematic approach for diagnosis and monitoring of VAP with clinical, laboratory, microbiological and LU data has recently been proposed in adults [12]. However, it has never been evaluated for diagnosis of VAP in neonates, especially in preterm infants with underlying CLD.

Our aim was to define clinical, laboratory, microbiological, chest X-ray and LU characteristics of VAP in our population and propose a multiparameter score for VAP diagnosis in neonates.

## Methods

This quality improvement project was part of the pre-implementation phase of a bundle of care aiming at reducing the number of VAP episodes and antibiotics use in our level III NICU at the Evelina London Children’s Hospital, London (UK). Infants with suspected VAP were prospectively identified between March 25, 2018, and May 25, 2019, by the attending medical team led by the NICU consultant using the a modified version of the previously published “NICU Lucerne” definition [5] of ventilation for more than 48 h and new start or change of antibiotic therapy due to worsening ventilation requirements (increased FiO2 by > 20%, increased pCO_2_ or ventilation demand) and at least one of the following: (i) clinical deterioration (temperature instability defined as axilla temperature of > 37.5 °C or < 36.5 °C that was deemed not to be due to environmental factors by the attending medical team, tachycardia, hypotension, increased frequency of episodes with bradycardias and/or desaturations), (ii) new or worsening opacity, consolidation or pleural effusion on chest X-ray, (iii) changes of tracheal secretions, (iv) abnormal laboratory parameters (C-reactive protein (CRP) > 10 mg/l, leucocytosis (white cell count > 20 × 10^9^/l) or leucopenia (white cell count < 5 × 10^9^/l)). Confirmed VAP was defined according to the above criteria plus isolation of a pathogenic microorganism in the airway aspirate.

Demographic, clinical, laboratory and microbiology data were collected from electronic patient records (BadgerNet®, Electronic Patient Records). Demographic data were collected on gestational age at birth and corrected age at VAP diagnosis, birth weight and actual weight. Clinical data included type of and reason for respiratory support, presence of respiratory deterioration, temperature instability within 24 h of VAP diagnosis and change in the amount and/or characteristics of airway secretions as recorded in the medical notes. Clinical data on risk factors known to be associated with VAP were also collected including number of intubation episodes, length of respiratory support with positive pressure ventilation and number of antibiotic courses. Laboratory data included CRP and white cell count at the time of VAP diagnosis and within the next 48 h. We collected semi-quantitative microbiology results and data on culture and sensitivity profile of pathogens that grew in the airway secretions.

We also applied the definition of Paediatric Ventilator-Associated Events (Ped-VAE), including Ventilator-Associated Condition (Ped-VAC), Infectious VAC (Ped-IVAC) and Possible Ventilator-Associated Pneumonia (Ped-PVAP) [13] to all cases to assess whether our definition for neonatal VAP were consistent with Ped-VAE, Ped-VAC, Ped-IVAC and/or Ped-PVAP.

Chest X-rays acquired as part of routine clinical care and stored on the Patient Archiving and Communication System were reported by the attending consultant radiologist. Reports were retrospectively reviewed by a neonatal consultant (VMP) to determine whether based on the CDC, ECDC, Lucerne and Dutch NICU definitions [14] of VAP, the following criteria were met: new emergence or worsening of consolidation, infiltration, pleural effusion or presence of cavitation or pneumatocele. Using the diagnosis on the radiological report, episodes were dichotomized based on whether the chest X-ray report was suggestive of VAP.

Additionally, LU scans were done within 24 h of VAP diagnosis as part of routine clinical care. LU scans were performed by trained members of the attending neonatal medical team using a GE Logiqe ultrasound machine (GE Healthcare®) with a L10–22 linear probe. The probe was held perpendicular to the ribs. For each hemithorax, three areas were defined by the anterior and posterior axillary lines (anterior, axillary and posterior). Anterior and posterior areas were divided into superior and inferior regions. At least nine standard views were acquired including right anterior apex and base, right axilla, right posterior apex and base, left anterior apex, left axilla, left posterior apex and base. A short clip was recorded in each view, downloaded anonymised and reviewed retrospectively offline by two neonatal consultants, NT and VMP with 1.5 and 6 years of experience respectively in performing and interpreting LU in neonates. In each view, the following parameters were assessed: aspect of the pleural line (presence of pleural sliding, thickness and regularity of the pleural line), presence of A-lines and B-lines, presence of consolidations and their characteristics (presence of static and/or dynamic air bronchograms, size and location in terms of translobar/non-translobar) and presence of pleural effusion. Translobar consolidation was defined as tissue-like image of the lung reflecting a loss of aeration with a regular inner limit (opposed visceral pleura). Non-translobar consolidation was defined as a tissue-like image limited by irregular “shredded” border as the non-aerated lung is in continuity with the aerated lung. When a consolidation was found, in some patients, it was assessed whether the consolidated area could be re-opened if indicated and possible by patient positioning and/or by increasing the mean airway pressure. For positioning, the consolidated area was positioned upward. For example, if a large consolidation was found in the posterior views, the patient was positioned prone for at least 1 h and the LU was repeated subsequently to assess re-opening of the consolidated area. Increasing mean airway pressure was achieved by stepwise increments of the positive end expiratory pressure and the peak inspiratory pressure, with close pressure-volume curve monitoring on the ventilator to detect over-distension [15].

Based on pathophysiological significance clinical, radiology and microbiology parameters [12] were identified and a scoring system for VAP diagnosis (multiparameter VAP score) was built post-hoc (Table 1). Clinical signs included respiratory deterioration, temperature instability and changes to airway secretions. In terms of radiological signs, for the purposes of scoring, on LU consolidations (> 0.5 cm depth), areas with linear/arborescent dynamic bronchograms and pleural effusion were included. The VAP score was calculated on Day 1 when antibiotics were started or changed and on Day 3 when microbiology result was available and antibiotic therapy was reviewed. To assess the clinical value of LU, the multiparameter VAP score was computed with and without LU findings on Day 1 and Day 3.

**Table 1:** Multiparameter ventilator-associated pneumonia score

	Day 1	Day3
Temperature instability		
No	0	0
Yes (axilla temperature < 36.5 °C or > 37.5 °C)	1	1
Change in airway secretions		
No	0	0
Yes (increased amount AND/OR change in colour/thickness of secretions that require more frequent suctioning)	1	1
Respiratory deterioration		
No	0	0
Yes (increased FiO2 by > 20% AND/OR need for escalation of respiratory support)	1	1
Lung ultrasound findings		
Presence of consolidation (> 0.5 cm)	2	2
Presence of pleural effusion	2	2
Presence of arborescence and/or linear dynamic bronchograms	2	2
Microbiology results of endotracheal aspirate (Day 3 only)		
No growth	N/A	0
Bacterial growth	N/A	1
Maximum score	9	10

A group of infants who received positive pressure respiratory support for at least 48 h and underwent LU scanning because of respiratory deterioration but did not have a VAP diagnosis was identified. Data were similarly collected and VAP scores calculated.

Data was also collected on presence of chronic pulmonary insufficiency of prematurity (CPIP), defined as respiratory morbidity after preterm birth during the birth hospitalization and through infancy and childhood [16], and CLD, defined according to National Institute of Health Consensus definition [17] and death.

### Statistical analysis

Statistical analyses were performed with IBM SPSS statistics 25.0 (SPSS Inc., Chicago, IL, USA). Distribution of continuous variables was determined. Independent samples t-test was used for comparison of normally distributed variables and non-parametric tests for independent samples for variables where the distribution significantly deviated from normal. Categorical variables were compared with Chi-square test. Continuous variables were reported as mean (standard deviation) or median (interquartile range) if normally or non-normally distributed, respectively. Categorical variables were reported as number (%). Statistical significance was assumed at *p* < 0.05.

Predictive values with 95% confidence intervals (CI) were calculated from 2 × 2 contingency tables, and area under the receiver operating characteristic curve (AUC) was determined. Multiple AUCs were computed to assess the predictive value of different approaches: clinical information only on Day 1, clinical information on Day 1 and chest X-ray, clinical information on Day 1 and LU, clinical information on Day 1 and microbiology result on Day 3 and finally clinical information on Day 1, microbiology result on Day 3 and LU. MedCalc was used to compute Youden’s J index for each AUC and to compare the AUCs between them [18].

Incidence of VAP was established over 1000 ventilator days. The number of ventilator days was determined during the study period using data recorded in BadgerNet® Electronic Patient Records.

Interclass correlation coefficient was calculated using SPSS to establish concordance between the two experts who reviewed the LU.

## Results

### Patients’ characteristics and outcome

**Fig. 1:**
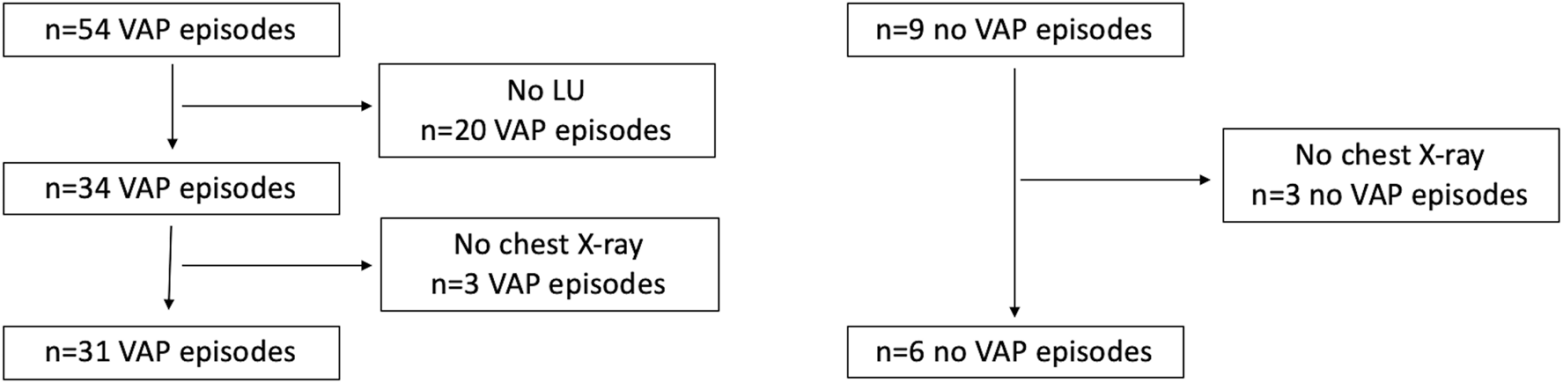
Flow diagram of episodes *LU* lung ultrasound, *VAP* ventilator associated pneumonia

We identified 54 VAP episodes in 28 infants. Fourteen infants had multiple VAP episodes and the mean number of relapses was 1.5 (standard deviation 1.6). Incidence of VAP was 10.47/1000 ventilator days. LU was not done in 20 episodes and chest X-ray in three. Therefore, we report 31 VAP episodes in 21 patients, who underwent LU and chest X-ray as part of their routine clinical care. The clinical characteristics were not significantly different between the episodes that were excluded and those that are included in the analysis. Nine episodes of respiratory deterioration that were deemed not to be due to VAP were identified in 8 patients. However, in three episodes no chest X-ray was done. Therefore, six episodes of respiratory deterioration in five patients were included that were not considered VAP. Flow diagram of the episodes is shown in Fig. 1.

In the VAP group, infants were on high frequency oscillation ventilation (HFOV) in 11 episodes. In five of these cases, the respiratory support was escalated from conventional assist control volume guarantee to HFOV at the time of respiratory deterioration. In one case, the infant was changed from HFOV to conventional ventilation. In 18 cases, the infants were on conventional assist control volume guarantee ventilation. In two episodes, the patients were on synchronized nasal intermittent positive pressure ventilation. Both of them were intubated at the time of respiratory deterioration. In the no-VAP group, the patients were on conventional ventilation in three episodes and on HFOV in two episodes. In one episode, the patient was on synchronized nasal intermittent positive pressure ventilation.

Main reasons for mechanical ventilation were respiratory distress syndrome (*n* = 8) [19], neonatal ARDS (*n* = 4 pulmonary haemorrhages, *n* = 1 congenital pneumonia *n* = 1 necrotizing enterocolitis) [20], sepsis-induced respiratory failure (*n* = 5) and pulmonary hypoplasia associated with oligohydramnios sequence (*n* = 2). The patient who was born at term had trachea-oesophageal atresia associated with Trisomy 18. In the no-VAP group, the main reasons for mechanical ventilation were respiratory distress syndrome (*n* = 2), sepsis induced respiratory failure (*n* = 2), congenital pneumonia (*n* = 1) and neonatal ARDS related to necrotizing enterocolitis (*n* = 1).

All but one infant in the VAP group had evolving CPIP at time of first VAP episode [16]. Out of 21 patients 5 died. Of the surviving infants, all had CLD at 36 weeks. In the no-VAP group, all five patients developed CPIP. Two of them died and the rest developed CLD at 36 weeks. Of note, all episodes were consistent with Ped-VAE. In the VAP group, two episodes, where the white cell count and temperature were within normal range, were consistent with Ped-VAC and the rest of the episodes with Ped-PVAP. In the no-VAP group, two episodes were classified as Ped-VAC.

Basic clinical characteristics of patients are shown in Table 2. Clinical and laboratory data at the time of respiratory deterioration are shown in Table 3.

**Table 2:** Patients’ basic clinical characteristics

	VAP *n* = 21 patients	No-VAP n = 5 patients	*p*
Gestational age (weeks^+days^) *	25^+2^ (2.8)	24 ^+ 2^ (1.33)	0.41
Female gender n (%)	7 (33%)	4 (80%)	0.07
Birth weight (grams) *	785 (320)	560 (110)	0.67
Complete course of antenatal steroids n (%)	16 (73%)	8 (80%)	0.96

*mean (standard deviation)

*n* number, *VAP* ventilator-associated pneumonia

**Table 3:** Patients’ clinical characteristics at respiratory deterioration

	VAP *n* = 31 episodes	No-VAP *n* = 6 episodes	*p*
Corrected age (weeks^+days^) *	31^+4^ (4.2)	29^+6^ (3.8)	0.43
Days at deterioration *	44.93 (28.57)	39 (31)	0.56
Weight at deterioration (grams) *	1463 (692)	1070 (350)	0.22
Days of ventilation (days) *	41.32 (29.48)	38 (32)	0.79
Number of intubation episodes *	5.35 (3.48)	4.3 (3.9)	0.34
Number of antibiotic courses *	4.48 (3)	3.3 (2.2)	0.36
Baseline FiO_2_ *	0.44 (0.2)	0.47 (0.32)	0.67
FiO_2_ at deterioration *	0.69 (0.24)	0.62 (0.26)	0.32
C-reactive protein at deterioration (mg/l) *	11.3 (12.57)	7.5 (2.12)	0.97
C-reactive protein repeat (mg/l) *	21.68 (39.88)	10.8 (14.44)	0.72
White cell count at deterioration (10^9^/l) *	27.15 (52.34)	17.2 (0)	0.62
White cell count repeat (10^9^/l) *	15 (10.37)	21 (10.74)	0.23

*mean (standard deviation)

*n number, VAP* ventilator associated pneumonia

### Clinical, laboratory and microbiology results

All patients had respiratory deterioration. FiO_2_ increased by more than 10% in 6 VAP episodes (19.3%) and by more than 20% in 20 episodes (64.5%). Temperature instability was reported in 23 episodes (74.2%). Changes to airway secretions, described as change in colour, amount and/or thickness that required more frequent suctioning were reported in all cases but one.

Leucocytosis at time of deterioration occurred in 6 (19.3%), whereas leukopenia in two (6.5%) episodes. CRP at time of deterioration was above 10 mg/l in 11 (35.4%) cases. There was no difference in white cell count (*p* = 0.27) or CRP (*p* = 0.18) taken at respiratory deterioration and repeated within 48 h.

One episode was defined as suspected and 30 as confirmed VAP. The patient whose microbiology culture of airway secretion showed no growth was already on antibiotics for 48 h prior to onset of suspected VAP. Microorganisms isolated from airway secretions are reported in Table 4. Of note, in three episodes more than one bacterium was isolated. Blood culture was positive in 3 episodes (*Escherichia coli n* = 1, *Klebsiella pneumoniae n* = 1, *Staphylococcus aureus n* = 1).

**Table 4:** Bacteria isolated from endotracheal tube secretions

Bacteria	*n*
*Klebsiella pneumoniae*	6
*Escherichia coli*	5
*Enterobacter cloacae*	5
*Staphylococcus aureus*	4
*Acinetobacter baumannii*	4
*Stenotrophomonas maltophilia*	3
*Pseudomonas aeruginosa*	2
*Proteus mirabilis*	2
Klebsiella aerogenes	2
*Citrobacter koseri*	2
*Chrysobacterium gleum*	1

### Radiology results

#### Chest X-ray

Chest X-ray was performed at the time of respiratory deterioration in 31 episodes in the VAP and in 6 episodes in the no-VAP group. Only 20 (64.5%) episodes met radiological criteria for VAP, exhibiting new appearance or worsening consolidations (*n* = 4 (12.9%)) and/or opacities (*n* = 15 (48.4%)) on chest X-ray. Two chest X-rays (33.3%) in the no-VAP group were described as worsening opacity (Table 5).

**Table 5:** Radiological findings (*n* (%))

	VAP *n* = 31	No-VAP n = 6	Pearson Chi-square asymptomatic significance 2-sided
Chest X-ray findings			
New emergence or worsening of consolidation/opacity	20 (64.5)	2 (33.3)	0.15
Pneumatocele/cavitation	0	0	N/A
Pleural effusion	0	0	N/A
Lung ultrasound findings			
Diffuse lung rockets	31(100)	6 (100)	N/A
Consolidations < 0.2 cm	27 (87.1)	6 (100)	0.35
Consolidation > 0.5 cm	31 (100)	4 (66.7)	0.001
Unilateral consolidation	6 (19.3)	2 (33.3)	0.45
Bilateral consolidation	25 (80.6)	2 (33.3)	0.02
Anterior consolidation	6 (19.3)	1 (16.6)	0.88
Posterior consolidation	22 (70.9)	2 (33.3)	0.08
Anterior and posterior consolidations	3 (9.6)	1 (16.6)	0.61
Non-translobar consolidation only	24 (77.4)	1 (16.6)	0.004
Translobar and non-translobar consolidation	7 (22.6)	3 (50)	0.17
Static bronchograms	31 (100)	5 (83)	0.02
Linear/arborescent dynamic bronchograms	23 (70.6)	2 (33.3)	0.05
Pleural effusion	3 (8.8)	0 (0)	0.43
Successful re-opening of consolidated area	8/11 (72.7)	2/2 (100)	0.16

*n* number, *N/A* not applicable, *VAP* Ventilator-associated pneumonia

### Lung ultrasound

LU was available for 31 VAP and 6 no-VAP episodes. The agreement in LU scoring was high between the two experts with an interclass correlation coefficient of 0.899 (95% CI 0.80–0.95).

In both the VAP and no-VAP groups, all patients had disseminated lung rockets in all lung fields (anterior, axillary and posterior). In the VAP group, there were 2 main groups of consolidations based on size, < 0.2 cm and > 0.5 cm (Online Resource 1). In all VAP episodes, there was at least one consolidation > 0.5 cm and in 70.9% there were 2 (22/31). Non-translobar consolidations were found in all episodes and both translobar and non-translobar consolidations in 7. Mean size of the non-translobar consolidation was 1.5 cm (range: 0.8–2.75). These were mainly seen posteriorly. Consolidations <0.2 cm were mainly located anteriorly (Online Resource 2). Static bronchograms were present in all episodes, linear/arborescent dynamic bronchograms (Online Resource 3) in 23 and pleural effusion in 3 (Table 5). Re-opening of the consolidated area was attempted in 11 episodes. Re-opening was not or only partially possible in 8 cases. The consolidated area was completely re-opened in 3 episodes (Online Resource 4 and 5).

LU was performed at the end of antibiotic treatment in 12 episodes. In 6 episodes, persisting consolidations suggestive of VAP were seen and all of these patients had a relapse within the next month. Among the 6 patients without LU findings suggestive of VAP at end of antibiotic treatment, only 1 relapsed within the next month.

In the no-VAP group, consolidations < 0.2 cm were present in all episodes. In four episodes, the consolidations were > 0.5 cm. Mean size of the non-translobar consolidations was 1.8 cm (range 1–2 cm). Static bronchograms were seen in all but one episode. This one episode was consistent with segmental atelectasis (Online Resource 6). Pleural effusion was not found in any of the patients, who did not have VAP. Re-opening of the consolidated area was attempted in two episodes and was successful in infants without VAP. Chest X-ray and LU findings are shown in Table 5.

### VAP score

**Fig. 2:**
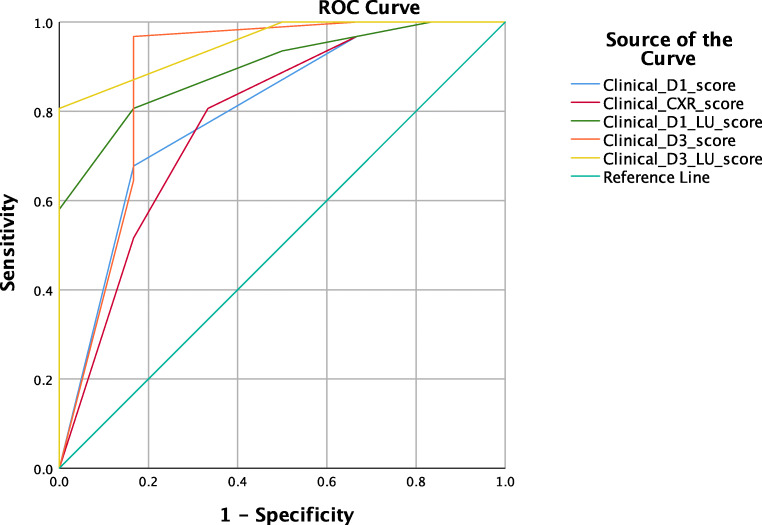
Area under the receiver operating curve for multiparameter ventilator -associated pneumonia score *CXR* chest X-ray, *D* day, *LUS* lung ultrasound

Predictive values of different approaches as described in the Methods were calculated and are shown in Table 6. A multiparameter VAP score of > 4 on Day 1 and > 5 on Day 3 gave the highest sensitivity (0.94) with AUC of 0.91 (standard error (SE) 0.06, 95% CI 0.8–1.00, *p* = 0.002) and 0.97 (SE 0.03, 95% CI 0.92–1.00, *p* = 0.0003), respectively (Fig. 2). Sensitivity and AUC showed that adding LU to clinical information improves the predictive value as opposed to combining clinical information with chest X-ray (Table 6). AUCs were compared, and following correction for multiple comparisons, no significant difference was found between them.

**Table 6:** Predictive values of multiparameter ventilator-associated pneumonia score on Day 1 and Day 3

	Clinical D1	Clinical D1 CXR	Clinical D1 LU	Clinical D3	Clinical D3 LU
Cut-off	2	3	4	2	5
Sensitivity	0.97	0.81	0.94	1	0.94
(95% CI)	(0.83–1)	(0.63–0.93)	(0.79–0.99)	(0.89–1)	(0.76–0.99)
Specificity	0.33	0.66	0.67	0.33	0.83
(95% CI)	(0.04–0.78)	(0.22–0.96)	(0.22–0.96)	(0.04–0.78)	(0.36–1)
Positive likelihood ratio	1.45	2.42	2.81	1.5	5.61
(95% CI)	(0.82–2.57)	(0.77–7.6)	(0.9–8.73)	(0.85–2.64)	(0.94–33.67)
Negative likelihood ratio	0.1	0.29	0.1	0	0.08
(95% CI)	(0.01–0.91)	(0.12–0.72)	(0.02–0.41)		(0.02–0.31)
Positive predictive value	0.88	0.93	0.93	0.88	0.97
(95% CI)	(0.91–0.93)	(0.8–0.97)	(0.82–0.98)	(0.81–0.93)	(0.83–0.99)
Negative predictive value	0.66	0.4	0.66	1	0.71
(95% CI)	(0.71–0.95)	(0.21–0.62)	(0.32–0.9)		(0.38–0.9)
AUC (SE)	0.88 (0.06)	0.85 (0.07)	0.91 (0.06)	0.99 (0.01)	0.97 (0.03)
p	0.004	0.008	0.002	0.0002	0.0003
Youden J index	0.68	0.51	0.6	0.95	0.79

*AUC* area under the receiver operating characteristic curve, *CI* confidence intervals, *CXR* chest X-ray, *D* day, *LU* lung ultrasound, *SE* standard error

## Discussion

We demonstrated that adding LU to clinical information could be an alternative to chest X-Ray to diagnose VAP in neonates. As there is no widely accepted consensus regarding definition of neonatal VAP, we have also introduced a multiparameter score comprising clinical, laboratory, microbiological and LU data that showed high predictive values for VAP diagnosis in neonates with underlying lung pathology.

Chest X-Ray has been the gold standard imaging method for diagnosing chest infection in paediatric patients, and it is a mandatory criterion in the most often applied VAP definitions, such as the CDC guidelines for infants ≤ 1 year and the ECDC criteria [5]. However, challenges in interpreting radiographic changes in patients with underlying lung pathology, like infants with CLD have been previously reported [5–7] and inter-rater reliability of chest X-ray interpretation was shown to be poor in this context [5]. In our series, only 64.5% of episodes met radiological criteria for VAP diagnosis based on chest X-ray. On the other hand, we found that LU had a high inter-rater reliability with only one disagreement between the two experts. Subpleural consolidation, consolidations > 1 cm, dynamic bronchogram, focal B-lines, pleural line abnormalities and pleural effusion have been shown to be characteristics of community-acquired pneumonia in hospitalized children with high specificity in suspected pneumonia [10, 11, 21, 22]. In adult VAP, subpleural consolidation and dynamic arborescent/linear air-bronchogram had high positive predictive values [23]. Of all the LU findings described in paediatric patients and adults, sub-pleural consolidations were also present in all our patients who did not have VAP and were thus not considered to be suggestive of VAP diagnosis. All patients in both VAP and no-VAP groups had diffuse lung rockets, related to the underlying lung pathology. We therefore excluded focal B-lines from our score. The LU findings that were suggestive of VAP in our patient population were consolidations > 0.5 cm accompanied by linear/arborescent dynamic bronchograms and/or associated with pleural effusion.

All LU in the VAP group showed large consolidations, which could either be atelectasis, collapse in dependant areas or VAP. Atelectasis could easily be recognized as a well limited area of consolidation with absent or very rare static bronchograms. Most challenging part of the assessment was to differentiate between areas of collapse and infection. Linear/arborescent dynamic bronchograms have been shown to be highly specific for pneumonia but identifying such changes in neonates with respiratory rates as high as 60/min and small tidal volumes could be challenging. Of note, identifying dynamic bronchograms was the only disagreement between the two experts in our cohort. Surprisingly, two infants in the no-VAP group had consolidations with linear dynamic bronchograms. Thus, specificity of linear/arborescent dynamic bronchograms in the collapsed dependant areas of infants with CLD needs further study. Punctiform dynamic bronchograms were identified in consolidations > 0.5 cm in both groups. However, these are not specific to pneumonia [23], rather a sign of de-recruitment. Recruitment of consolidated areas was another criterion to differentiate between collapsed areas and VAP. We hypothesised that when a consolidation cannot be completely recruited either by positioning of the infant or by recruitment airway manoeuvres, this would be suggestive of VAP. Most patients in the VAP group had consolidations that partially re-opened, changing from a consolidated area to white lungs following recruitment manoeuvres. Partial re-opening may be related to the presence of collapsed areas around areas of infection. All consolidations in the control group were possible to recruit.

LU to monitor recovery following VAP and efficacy of antibiotics was previously studied [24]. In our cohort, all patients (6/12) who had persisting consolidation suggestive of VAP at the end of antibiotic treatment (without any other clinical symptoms) had a relapse within the next month, whereas only 1/6 patients without LU findings suggestive of VAP at end of treatment relapsed. Performing LU at end of antibiotic treatment was not standard of care during this pre-implementation phase, explaining the small number of episodes where such assessment was performed. A LU score to quantify the global loss of aeration has been proposed in adult VAP patients. Its modification in days showed a significant correlation with re-aeration as assessed by quantitative CT scan (rho = 0.85) when computed after 7 days of antibiotics [25]. LU to monitor response to antibiotics treatment in neonates should be investigated further as optimal duration for treatment is currently unknown.

Despite LU being a promising tool to aid VAP diagnosis, similar to other neonatal respiratory pathologies [26], there is no single highly specific marker of VAP in neonates. Clinical and laboratory findings have been shown to be nonspecific [3, 25]. In our series, only 26% of infants had abnormal white cell count and only 41.8% had increased CRP. Other markers, such as procalcitonin have no positive impact on the accuracy of clinical and ultrasound-based scores for VAP diagnosis [27]. Microbiological samples cannot guide early decisions on starting antibiotics. A multiparameter systematic approach for diagnosis and monitoring of VAP with clinical, laboratory, microbiological and LU data is essential. A multiparameter score has recently shown to have greater specificity and sensitivity than clinical signs alone in adults [12]. To our knowledge, this is the first time that a multiparameter score combining clinical, microbiological and LU characteristics has been studied in neonates. Furthermore, sensitivity, specificity and AUC showed that predictive values increased when LU was included in the multiparameter VAP score.

Incidence of VAP in our NICU was 10.47/1000 ventilator days, which is in line with published incidence of 2.7–11.8/1000 ventilator days in developed countries [5]. Recent studies have reported lower incidence of VAP after implementation of VAP prevention bundles that combined antimicrobial stewardship and infection control measures, such as hand hygiene, isolation guidelines, handling and disinfection of patient care equipment, instruments and devices and use of personnel protective equipment [5, 28, 29]. This reduction had a significant impact on antibiotic use.

The main limitation of our cohort is its small sample size. As a result, it was not possible to undertake multivariate analysis adjusting for cofounders. Also, there is a mismatch between the size of the group of patients with and without VAP. We prospectively identified patients over a 14 months period during the pre-implementation phase of a VAP prevention care bundle. However, some potentially eligible patients might have been inevitably missed in context of non-trial settings. Furthermore, although LU is routinely performed on our NICU since 2018, experience and confidence of physicians, especially of those in training, performing LU varies; hence, LU was not available in every episode. Recruitment manoeuvre, although a dynamic and important element to explore further, was attempted only in a small number of episodes.

In summary, LU is a potential diagnostic tool in neonatal VAP. Compared with CXR, we found LU to be more useful to differentiate areas of atelectasis from areas of de-recruitment or areas of pneumonia, to assess the effect of positioning and recruitment manoeuvres on large consolidations and potentially to monitor evolution of consolidations following antibiotic treatment. However, this needs to be explored further in larger studies. While the proposed multiparameter VAP score, incorporating LU findings, showed promising predictive values for diagnosis of VAP in neonates with underlying CLD, it needs further testing in larger patient populations under prospective, multi-centre clinical trial settings.
